# Temperature-Frequency–Dependent Viscoelastic Properties of Neat Epoxy and Fiber Reinforced Polymer Composites: Experimental Characterization and Theoretical Predictions

**DOI:** 10.3390/polym12081700

**Published:** 2020-07-29

**Authors:** Kakur Naresh, Kamran Ahmed Khan, Rehan Umer, Alagumalai Vasudevan

**Affiliations:** 1 Department of Aerospace Engineering, Khalifa University of Science and Technology, Abu Dhabi 127788, UAE; rehan.umer@ku.ac.ae; 2 Department of Mechanical Engineering, Saveetha School of Engineering, Chennai 602105, India; vasudevana.sse@saveetha.com

**Keywords:** laminate, mechanical properties, dynamic mechanical analysis, viscoelastic properties, glass transition temperature, activation energy

## Abstract

In general, aerospace structures manufactured using fiber reinforced polymer composites are exposed to fluctuating temperatures and subjected to cyclic loading during their service life. Therefore, studying the temperature-frequency dependent properties of composites for different fiber orientations is essential. However, such experiments are expensive, time-consuming and labor-intensive while theoretical models minimize these issues, but temperature-frequency-dependent viscoelastic models for predicting the full-range of the storage and loss moduli curves of composites are limited. In this study, the dynamic mechanical properties of a neat epoxy resin, unidirectional ([0°]_6_, [45°]_6_ and [90°]_6_), symmetric angle-ply [+45°/−45°/+45°]_s_ and quasi-isotropic [±45°/0°/90°]_s_ carbon/epoxy and glass/epoxy composite panels were investigated. Experiments were performed from room temperature (approximately 35 °C) to 160 °C at five different frequencies (1, 10, 20, 33 and 50 Hz). Two parameter viscoelastic models as function of temperature and frequency were used, and their applicability in predicting the storage and loss moduli for the entire region of the temperature curve is shown. The storage modulus values were compared and validated against the static flexural modulus values coupled with scanning electron microscopy analysis. The flexural and storage moduli values were found to be higher for [0°]_6_ carbon/epoxy composites, while the activation energy values were found to be higher in the case of [+45°/−45°/+45°]_s_ carbon/epoxy composites compared with epoxy resin and other laminates in different orientations. The predicted results were in reasonably good agreement with the experiments. Both experimental and modeling approaches used in this study are highly valuable for designing aerospace composites for harsh in-service loading conditions.

## 1. Introduction

The use of fiber reinforced polymer composites (FRPCs) for liquid propellant rockets [1], aircraft landing gear components and jet and turboprop engine cowlings [2] has been increasing in recent times. In particular, carbon/epoxy and glass/epoxy composites are nowadays being widely used for automobile drive shafts, helicopter rotor blades, offshore ship applications, active and passive damping technologies [3,4]. This is due to their excellent corrosion resistance, light weight, high strength and stiffness, high natural frequency and better damping properties compared to conventional metallic materials [5,6]. However, the strength and stiffness of FRPCs can drastically decrease above the glass transition temperature (T_g_) of the polymer [7]. On the contrary, it is well known that metallic components possess superior thermal properties than FRPCs [8]. Grigoriou and Mouritz [9] reported that the surface plies of carbon/epoxy composites absorb heat much more rapidly than aluminum alloys. However, the heat flow through the thickness direction is very slow for the former, which might be attributed to its lower thermal conductivity. Moreover, carbon/epoxy composites retain their mechanical properties (strength and stiffness) over a wider range of temperatures than aluminum alloys. In a later study, the authors [10] concluded that the quasi-isotropic (QI) laminates having 45° surface plies and 0° middle plies possess higher thermal surface protection than those of 0° surface plies and 45° middle plies. 

There is research in progress to replace aluminum alloys with FRPCs for high-temperature applications owing to many reasons. The thermal behavior of FRPCs relies on many parameters, such as the type of fiber and matrix used, and their fiber volume fraction, orientation, stacking sequence, temperature, frequency, etc., [11,12,13,14]. More importantly, the thermal properties of FRPCs vary with the type of manufacturing process. Khan et al. [15] found higher T_g_ values in aerospace graded carbon/epoxy composite panels manufactured by quickstep processing owing to increased cross-linking, compared to T_g_ values of such panels manufactured by autoclave processing.

The failure mechanisms are different for composites containing different fiber orientations [16]. Therefore, selecting the appropriate lay-up orientation for the desired application is critical for designers, though the fiber orientation effect saves the material wastage by eliminating the fibers in a particular direction [17,18]. The transverse ply (90°) cracking is a major concern for FRPCs for high-temperature applications. The detection of these cracks is difficult during the NDT assessment. NASA X-33 launch vehicle operation was canceled due to leakage found in the pressure vessel due to transverse ply cracks. These cracks can cause primary leakage in pressure vessels at cryogenic temperatures and delamination at high temperatures [19,20,21]. However, the advantage of using QI laminates for most of the high-temperature applications is that they are less affected by stress concentrations caused by transverse plies [22]. QI and angle-ply laminates possess pseudo-ductility features in all-fiber directions, as a result, the catastrophic failure of the composite can be reduced [23,24]. The pseudo-ductility effect can change the failure mode from delamination to fiber breakage. The laminates fabricated with (±45°) plies can possess high fracture toughness and delay the crack propagation [25,26]. However, high fracture toughness is a desired property for the time-temperature-frequency dependent behavior of materials. Hence, the fabrication of QI laminates with (±45°) plies is desirable, particularly placing 45° plies on the outer surface improves structural performance.

The Ground Vibration Test (GVT) is an important test to certify new or heavily modified aircraft, which can be performed at different frequencies to investigate the dynamic aero-elastic behavior [27]. Dynamic Mechanical Analyzer (DMA) is a widely used instrument for characterizing the time-temperature-frequency dependent behavior of materials. The input data can be obtained with the aid of a DMA to generate the frequency-dependent numerical models for GVT. FRPCs possess excellent vibrational properties and usually can avoid resonance conditions, which depend on fiber orientation, viscoelastic properties and the T_g_.

In the past, many researchers [28,29,30,31,32] used DMA for characterizing the viscoelastic behavior of polymers and their composites. However, only a few studies are available in the literature on the dynamic mechanical properties of FRPCs containing different fiber orientations [13,33,34,35,36]. Bergent et al. [37] recently studied the dynamic mechanical properties of woven roving carbon/epoxy composites of cross-ply and QI configurations, over a temperature range of 30 °C to 220 °C, and the frequency range of 1 Hz to 50 Hz. The activation energies were estimated using the Arrhenius model. The activation energy values were reported to be higher for cross-ply laminates than QI laminates. It was also found that the T_g_ increases up to 14 °C within the frequency range of 1 Hz and 50 Hz. Guo et al. [34] correlated the static flexural modulus of 0° and (0°/90°) with dynamic mechanical properties using DMA, equipped with three-point bending fixture over a temperature range from room temperature to 120 °C, and at a constant frequency of 1 Hz. They developed a single parameter viscoelastic model based on kinetic parameters [38] for predicting the complete history of storage modulus and found good agreement with the experiments. In a recent study, Xu and Gupta [39] introduced a novel approach to estimate the strain rate effect at different temperatures on the elastic modulus of ethylene-vinyl acetate using frequency-domain dynamic mechanical properties. They have used a radial basis neural network to obtain the strain rate dependent mechanical properties from the frequency-dependent viscoelastic properties. The studies related to frequency or strain rate sensitivity of carbon/epoxy composites are not clear. Some researchers reported that carbon/epoxy composites are insensitive to rate effects [40] while others [3] reported that carbon/epoxy composites are less sensitive to rate effects, compared to glass/epoxy composites. In a recent study [4], it was found that the strain rate sensitivity of carbon/epoxy composites relies on fiber orientations. Further studies are required to fill this research gap.

In this study, the dynamic mechanical properties of neat epoxy and different fiber orientations of carbon/epoxy and glass/epoxy composites are investigated experimentally and theoretically, by performing several parametric studies using temperature-frequency dependent viscoelastic models. A new type of symmetric angle-ply laminate (45°/−45°/45°)_s_ for high-temperature applications was introduced in this study, in contrast to conventional (±45°)_s_ laminates. The effect of frequency on storage modulus is investigated using a frequency-dependent empirical equation. The effect of frequency on both the loss modulus and loss factor T_g_ values are investigated using the Arrhenius model. Mechanical (flexural, tensile and Izod impact) properties of previous studies [21,41,42] are used to compare the dynamic mechanical properties of the present study. Also, tensile properties are used to determine the interfacial strength indicator value of the composite. The room temperature flexural modulus values are correlated with initial storage modulus values. 

## 2. Materials and Methods

### 2.1. Materials

**Figure 1:**
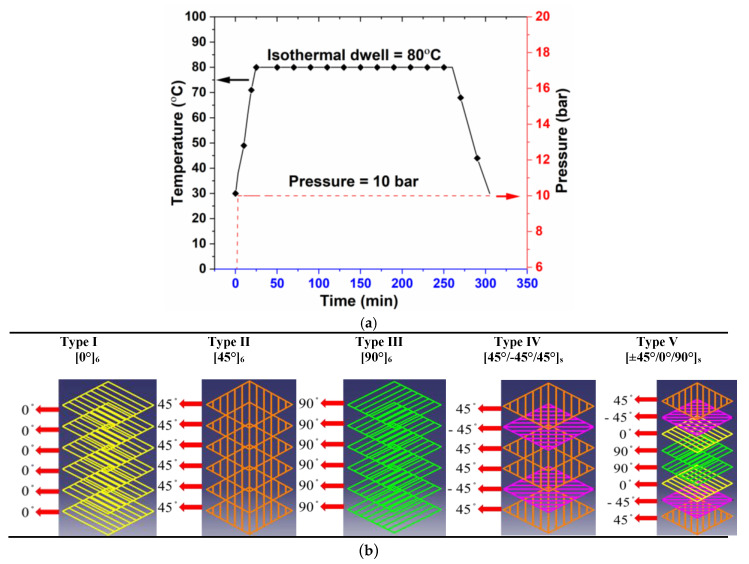
(**a**) Cure cycle used in this study to fabricate the laminates. (**b**) Schematic representation of different fiber orientations of FRPCs.

In this study, unidirectional carbon fabrics of 400 g/m^2^ and glass fabrics of 450 g/m^2^ were used as reinforcements, and epoxy resin (LY 556) supplied by Sigma-Aldrich (Chennai, Tamil Nadu, India) was used as the resin system for fabricating different fiber orientation laminates ([0°]_6_, [45°]_6_, [90°]_6_, [45°/−45°/45°]_s_ and [±45°/0°/90°]_s_) using the compression molding technique. The motivation behind choosing of 45° plies as surface plies in symmetric angle ply and quasi-isotropic laminates was mainly for enhancing the structural integrity [10,24,43]. The samples were manufactured according to the manufacturer’s recommended cure cycle. Temperature and pressure profiles used for fabricating the laminates are given in Figure 1a. Temperature used in the dwell region was 80 °C for 4 h. A constant pressure of 10 bar was maintained throughout the cycle. All laminates were post cured at room temperature for 24 h. 

These composite panels are schematically represented in Figure 1b. Fiber volume fractions of the laminates with the range of 40% to 54% obtained in this study, which are given in Table 5. Neat epoxy resin specimens were made using a resin casting technique in a mold with a cavity of 3 mm.

### 2.2. Experimental Details

#### 2.2.1. Static Mechanical and Impact Studies

Flexural, tensile and notched Izod impact tests were performed in our previous studies [21,41,42], according to ASTM D790, D3039 and D256, respectively. The dimensions such as length and width used were 125 mm × 12.7 mm for flexural tests with a span length of 50 mm and for tensile tests these values were 250 mm × 20 mm. Flexural and tensile tests were performed at the displacement rate of 5 mm min^−1^ using an Instron universal testing machine and a FIE universal testing machine, respectively. An EE-2 clip-on extensometer was placed in the gauge portion of the specimens and recorded the displacement of the specimens during tensile loading. The dimensions of the notched impact tests used were 63 mm of length × 12.7 mm of width. 

#### 2.2.2. Scanning Electron Microscopy (SEM) Studies

The fractured surfaces of three-point bending tested specimens of neat epoxy, glass/epoxy and carbon/epoxy composites were investigated at the voltage of 10 kV using a scanning electron microscope (EVO MA 15, ZEISS, Germany)). These specimens were gold-coated before SEM for 120 s, using an ion sputter instrument. This is for increasing the electrical conductivity of the specimens and thus to avoid charging of the specimens during analysis. SEM micrographs were captured at the magnification of 300×.

#### 2.2.3. Dynamic Mechanical Analysis

**Figure 2:**
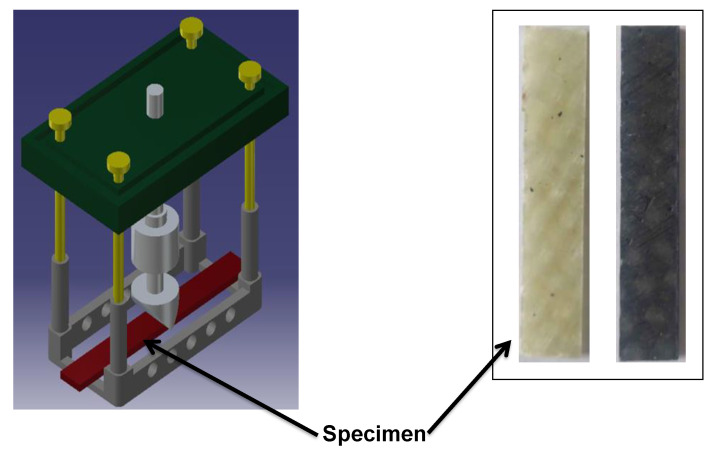
Three-point bending fixture with rectangular specimens of glass/epoxy and carbon/epoxy composites.

A DMA 242 E instrument (NETZSCH, Ahlden, Germany) was used in three-point bending mode for performing the temperature-frequency dependent dynamic mechanical experiments. The neat epoxy and different fiber orientation composite specimens were subjected to a temperature ramp from atmospheric temperature (~35 °C) to 160 °C, at five different frequencies, namely 1, 10, 20, 33 and 50 Hz. Liquid nitrogen was not used since all tests were performed from atmospheric temperature to positive elevated temperatures (up to 160 °C). The experiments were performed on a rectangular specimen having a width and span of 11.2 mm and 42 mm respectively, at a constant applied load of 12 N, with a displacement amplitude of 100 μm. The thickness of the samples ranged from 3 mm to 3.1 mm for composite plates having six layers, and 3.3 to 3.4 mm for eight layer laminates. The samples were heated at a rate of 3 °C/min. Figure 2 shows a schematic representation of a three-point bending fixture used for DMA studies. 

## 3. Theoretical Modeling

Experimental studies are expensive, time-consuming and require manpower while theoretical studies reduce the cost, time and manpower. Therefore, further theoretical studies are required for characterizing the vibration performance of different fiber orientations of FRPCs [44]. In this section, the challenges associated with the existing theoretical models and the better possible ways to enhance those challenges are discussed.

### 3.1. Viscoelastic Properties:

The vibro-acoustics response of viscoelastic material relies on the delay between the input strain and the output stress, as a function of applied frequency [45,46]. The dynamic modulus $E_{d}\left(\omega \right)$ of viscoelastic material is a function of the storage modulus and loss modulus, which is written as:

(1) $$E_{d}\left(\omega \right)=\;E^{\prime }\left(\omega \right)\pm i\;E\prime \prime \left(\omega \right)$$

where $E^{\prime }\left(\omega \right)$ and E″(ω) are the storage and loss moduli, respectively. The storage modulus is the essential viscoelastic parameter which determines the elastic response of the viscoelastic material during mechanical loading. Storage modulus is a function of both temperature and frequency. Storage modulus at room temperature is considered as the initial or maximum storage modulus in this study. When the viscoelastic material is subjected to a sinusoidal load for a longer time, the sample loses its mechanical energy. The amount of mechanical energy dissipated as heat in the viscoelastic material is called as loss or viscous modulus. This loss modulus represents the viscous response of the material. The amount of damping depends on the ratio of  E″(ω) and $E^{\prime }\left(\omega \right)$ values, which is called as loss tangent and given by:

(2) $$\tan\delta =\frac{E\prime \prime \left(\omega \right)\;}{E^{\prime }\left(\omega \right)}$$

**Figure 3:**
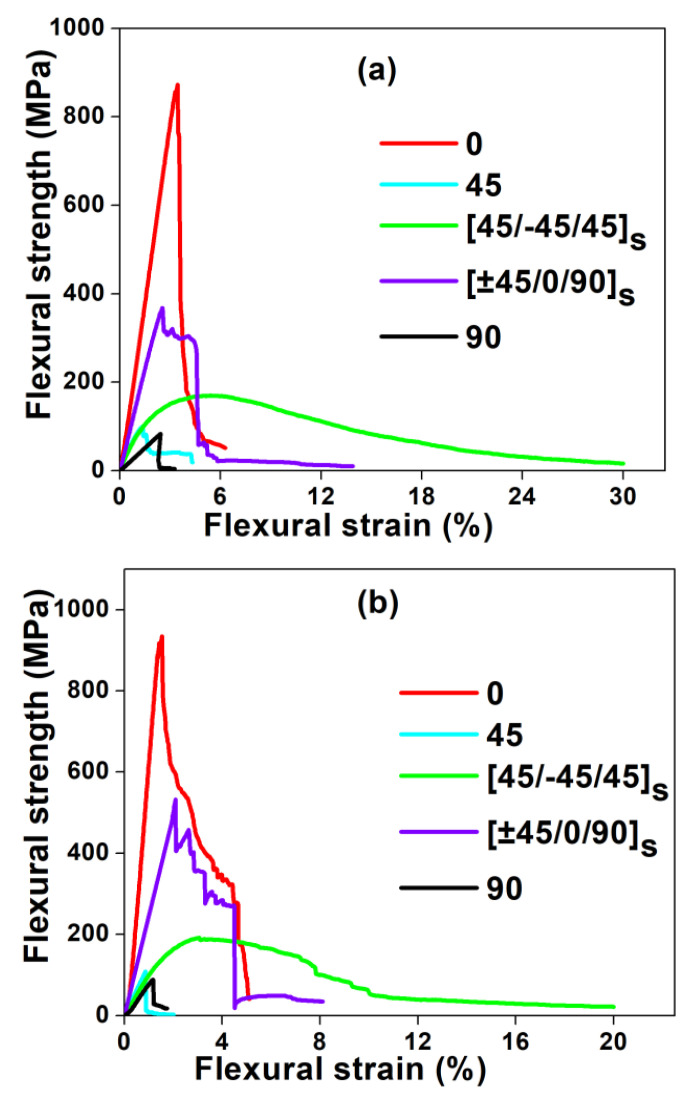
Flexural properties of composites: (**a**) GFRP and (**b**) CFRP.

The viscoelastic properties of FRPCs follow three regions such as glassy (β), transition (α) and rubbery (γ). The magnitude of the drop in properties is higher in the transition (α) region compared to glassy region. This is due to changes in molecular state, from immobile at room temperature to mobile in the transition region. The mobility of molecular chains further increases in the rubbery region, where the curve is a plateau, which can be clearly seen in Figure 3. The glass transition temperature can be divided into three types, namely T_g_ (E’)_middle_ or T_mg_, T_g_ (E”)_max_ and T_g_ (tan δ)_max,_ corresponding to the inflection point of E’, peak values of E” and tan δ. Out of all three glass transition temperature values, T_g_ (E’)_middle_ is the least and T_g_ (tan δ)_max_ is the highest. These glass transition temperature values are the functions of the applied frequency. Arrhenius model connects the frequency with the activation energy and glass transition temperature [47,48], which can be written as:

(3) $$\ln\;\left(f\right)=-\left(\frac{E_{a}}{{\mathrm{RT}}_{g}}\right)+\ln\;\left(P\right)$$

where $f,{\;E}_{a},\;R$ and P are the frequency, activation energy, gas constant and pre-exponential factor, respectively. Henceforth, the activation energy is directly proportional to the slope of $\ln\;\left(f\right)\;\mathrm{vs}.\;1/\left(T_{g}\right)$ from Equation (3) [49], which can be given by:

(4) $$E_{a}=-R\;\frac{d\;(\ln\;f\;)}{d\;(\left(1/T_{g}\right)}$$

#### 3.1.1. Modeling Approaches for Predicting the Storage and Loss Moduli

As mentioned earlier, the viscoelastic properties of polymers and FRPCs depend on temperature and frequency. Therefore, it is essential to use a theoretical model that accounts for these effects. Drozdov [50] proposed a temperature-dependent model to estimate the instantaneous storage modulus $\left(E_{i\;}^{\prime }\right)$**.** However, this model is capable to predict the $\;E^{\prime }$ in the glassy region but not to capture the rubbery plateau. Mahieux et al. [51,52] developed the temperature-dependent statistical model (based on the Weibull parameter (w_i_)) to predict the $E_{i\;}^{\prime }$ for the whole range of curve, which can be written as:

(5) $$\;E^{\prime }\left(T\right)=\left(E_{1}^{\prime }-E_{2}^{\prime }\;\right){\;e}^{-{\left(\frac{T}{T_{\beta }}\right)}^{w_{\beta }}}+\left(E_{2}^{\prime }-E_{3}^{\prime }\;\right){\;e}^{-{\left(\frac{T}{T_{\alpha }}\right)}^{w_{\alpha }}}+\left(E_{3}^{\prime }\;\right){\;e}^{-{\left(\frac{T}{T_{\gamma }}\right)}^{w_{\gamma }}}$$

However, the effect of frequency was not considered in this model. Later, Richeton et al. [53] modified the Equation to consider both the temperature and frequency effects which can be given by,

(6) $$\;E^{\prime }\left(T,\;f\right)=[\left(E_{1}^{\prime }\;\left(f\right)-E_{2}^{\prime }\;\left(f\right)\right){\;e}^{-{\left(\frac{T}{T_{\beta }\;\left(f\right)}\right)}^{w_{\beta }}}+\left(E_{2}^{\prime }\;\left(f\right)-E_{3}^{\prime }\;\left(f\right)\right){\;e}^{-{\left(\frac{T}{T_{\alpha }\;\left(f\right)}\right)}^{w_{\alpha }}}+\left(E_{3}^{\prime }\;\left(f\right)\right){\;e}^{-{\left(\frac{T}{T_{\gamma }\;\left(f\right)}\right)}^{w_{\gamma }}}]$$

where $E_{1}^{\prime }\;\left(f\right)$ is the initial storage modulus at the starting point of $\;E^{\prime }\left(T,\;f\right)$ curve, $E_{2}^{\prime }\;\left(f\right)$ and $E_{3}^{\prime }\;\left(f\right)$ are the instantaneous storage moduli at the beginning of the transition and rubbery regions, respectively. $T_{\beta }\left(f\right),{\;T}_{\alpha }\;\left(f\right){\;\mathrm{or}\;T}_{g}\left(f\right){\;\mathrm{and}\;T}_{\gamma }\;\left(f\right)$. are the transition temperatures in the β, α and γ regions. $w_{\beta },{\;w}_{\alpha }\;$and $w_{\gamma }$ are the Weibull moduli for the corresponding regions. These statistical parameters can be estimated through a nonlinear least square method using a trust-region algorithm. The procedure to estimate the Weibull moduli are reported elsewhere [54]. The Weibull moduli remain the same as in Equation (5) while the change was made by considering the frequency effect into the parameters $E_{i\;}^{\prime }\;\left(f\right)$ and $T_{i}\;\left(f\right),\;i.e.,{\;T}_{\beta }\left(f\right),{\;T}_{\alpha }\left(f\right){\;\mathrm{and}\;T}_{\gamma }\;\left(f\right):$

(7) $$\;E^{\prime }\left(f\right)={E^{\prime }}^{\mathrm{ref}}\;\left(f\right)\;\left(1+\mathrm{mlog}\;(\frac{f}{f^{\mathrm{ref}}}\right))$$

(8) $$\frac{1}{T_{\beta }\left(f\right)}=\frac{1}{T_{\beta }{}^{\mathrm{ref}}}+\frac{k}{{\left(E_{a}\right)}_{\beta }}\ln\;(\frac{f^{\mathrm{ref}}}{f})$$

(9) $$T_{\alpha }\left(f\right)={\;T}_{\alpha }{}^{\mathrm{ref}}+\frac{-C_{2}{}^{\mathrm{ref}}\log\;(\frac{f^{\mathrm{ref}}}{f})\;}{C_{1}{}^{\mathrm{ref}}+\;\log\;(\frac{f^{\mathrm{ref}}}{f})}$$

(10) $$T_{\gamma }\;\left(f\right)={\;T}_{\gamma }{}^{\mathrm{ref}}\;\left(1+0.01\log\;(\frac{f}{f^{\mathrm{ref}}}\right))$$

where ${\;E^{\prime }\;}^{\mathrm{ref}},{\;T}_{\beta }{}^{\mathrm{ref}},T_{\alpha }{}^{\mathrm{ref}}{\;\mathrm{and}\;T}_{\gamma }{}^{\mathrm{ref}}\;$are the storage modulus and transition temperatures in the β, α and γ regions, respectively at the reference frequency ($f^{\mathrm{ref}})$ of 1 Hz. These reference values can be obtained from DMA experiments. ${\left(E_{a}\right)}_{\beta }$ is the activation energy corresponding to $T_{\beta }\;\left(f\right)$. k is the Boltzmann constant. $C_{1}{}^{\mathrm{ref}}$ and $C_{2}{}^{\mathrm{ref}}$ are the Williams-Landel-Ferry (WLF) model parameters [55]. m is the frequency sensitivity coefficient. Though this model (Richeton et al. [53]) has great potential to predict the temperature-frequency dependent properties, it requires several instantaneous storage modulus values as inputs from the experimental curve. Gu and Asaro [56] proposed a simple temperature-based empirical power-law model. However, in this model, the storage modulus is assumed to be zero when the curve reaches the reference temperature. In the actual cases, the storage modulus never becomes zero.

Though Einstein’s and Nielson models [13,49,57] predict well the viscoelastic properties of short fiber and nanofiller composites, these models are not so effective to capture the fiber orientation effect on viscoelastic properties (storage modulus and loss factor or loss tangent) since the only variable in these models are fiber volume fraction. Henceforth, other models are required to predict the viscoelastic behavior of FRPCs for different fiber orientations. Also, the above- discussed models are only for predicting the storage modulus. However, it is vital to predicting the instantaneous loss modulus $\left(E_{i}^{"}\right)$ as well since the temperature corresponding to loss modulus and loss tangent peak values are considered as the T_g_ in most other research work [13,49]. The loss tangent curve can be predicted from $E_{i\;}^{\prime }$ and $E_{i}^{"}$ curves.

Feng et al. [36,37] developed the two-parameter viscoelastic model for predicting the storage and loss modulus and T_g_ values. The model predictions were validated with the experiments performed on epoxy and glass/epoxy composites of different fiber orientations using three-point bending, single and dual cantilever fixture and, obtained a good agreement. 

The degree of glass transition $\left(\alpha_{T_{g}}\right)$ can be expressed in terms of storage, glassy and rubbery modulus values, which can be written as [38]:

(11) $$\left(\alpha_{T_{g}}\right)=\frac{E_{G}^{\prime }-E_{i\;}^{\prime }}{E_{G}^{\prime }-E_{R}^{\prime }}$$

where $E_{G}^{\prime }$ and $E_{R}^{\prime }$ are the glassy and rubbery storage modulus, respectively. Equation (11) can be written as

(12) $$\;E^{\prime }\left(T,\;f\right)={\;E}_{G}^{\prime }-\left(\alpha_{T_{g}}\right)\;\left(E_{G}^{\prime }-E_{R}^{\prime }\right)$$

In order to consider the temperature effect, the two parameters, namely L(f) and S(f) are introduced in Equation (11) and can be written as [35]:

(13) $$\frac{{d\alpha }_{T_{g}}}{\mathrm{dT}}=\;L\left(f\right)\;\left(1-\alpha_{T_{g}}^{S\left(f\right)}\right){\;\alpha }_{T_{g}}$$

where L(f) and S(f) are the intrinsic growth rate and the symmetry of the glass transition region, respectively. When $\underset{T\to T_{\mathrm{mg}}}{\begin{array}{c} Limit \end{array}}\alpha_{T_{g}}=0.5\;$, therefore, the solution of Equation (13) is given by:

(14) $$\left(\alpha_{T_{g}}\right)=\frac{1}{{\left[\left(2^{S}-1\right)e^{L\left(f\right)\;S\left(f\right)\;\left(T_{m}{}_{g}\left(f\right)-T\right)}+1\right]}^{\left(\frac{1}{S\left(f\right)}\right)}\;}$$

Substituting Equation (14) into Equation (12) and is given by:

(15) $$\;E^{\prime }\left(T,\;f\right)={\;E}_{G}^{\prime }-\frac{E_{G}^{\prime }-E_{R}^{\prime }}{{\left[\left(2^{S\left(f\right)}-1\right)e^{L\left(f\right)\;S\left(f\right)\;\left(T_{m}{}_{g}\left(f\right)-T\right)}+1\right]}^{\left(\frac{1}{S\left(f\right)}\right)}\;}$$

When S(f) = 1, $\left(\alpha_{T_{g}}\right)=\frac{1}{\left[e^{L\left(f\right)\left(T_{m}{}_{g}\left(f\right)-T\right)}+1\right]\;}$, therefore, Equation (15) becomes:

(16) $$\;E^{\prime }\left(T,\;f\right)={\;E}_{G}^{\prime }-\frac{E_{G}^{\prime }-E_{R}^{\prime }}{\left[e^{L\left(f\right)\left(T_{m}{}_{g}\left(f\right)-T\right)}+1\right]}$$

Similarly, the loss modulus can be written in terms of temperature [36]:

(17) $$\;E^{\prime }\left(T,\;f\right)=\;C\;\frac{L\left(f\right)\;\left(2^{S\left(f\right)}-1\right)e^{L\left(f\right)\;S\left(f\right)\;\left(T_{m}{}_{g}\left(f\right)-T\right)}}{{\left[\left(2^{S\left(f\right)}-1\right)e^{L\left(f\right)\;S\left(f\right)\;\left(T_{m}{}_{g}\left(f\right)-T\right)}+1\right]}^{\left(\frac{1}{S\left(f\right)}+1\right)}\;}$$

where $C=\frac{\left(\;E_{\prime \prime }\right)\max}{{\left[\frac{L\left(f\right)\;S\left(f\right)}{S\left(f\right)+1}\right]}^{\left(\frac{1}{S\left(f\right)}+1\right)}\;}$; L(f), S(f) and $T_{m}{}_{g}\left(f\right)$ are the functions of the frequency. The linear relationship between $\ln\;\left(f\right)\;\mathrm{vs}.\;\frac{1}{T_{g}}\;$ is established from the Arrhenius model. Similarly, the linear relationship between $\ln\;\left(f\right)\;\mathrm{vs}.\;\frac{1}{L\left(f\right)}$ and $\ln\;\left(f\right)\;\mathrm{vs}.\;\frac{1}{S\left(f\right)}$ are written as [35,36]:

(18) $$\ln\;\left(f\right)=A_{1}\frac{1}{L\left(f\right)}+\ln\;\left(B_{1}\right)$$

(19) $$\ln\;\left(f\right)={\;A}_{2}\frac{1}{S\left(f\right)}+\ln\;\left(B_{2}\right)$$

where $A_{1}$ and $\ln\;\left(B_{1}\right)$ are the slope and intercept, respectively of the curve $\ln\;\left(f\right)\;\mathrm{vs}.\;\frac{1}{L\left(f\right)}$; Similarly, A_2_ and $\ln\;\left(B_{2}\right)$ are the slope and intercept, respectively of the curve $\ln\;\left(f\right)\;\mathrm{vs}.\;\frac{1}{S\left(f\right)}$; Interestingly, the values $\frac{1}{L\left(f\right)}\;\mathrm{and}\frac{1}{S\left(f\right)}$ of epoxy, glass/epoxy and carbon/epoxy vary linearly with the frequency, which can be seen in Table 1 and Table 2, respectively.

#### 3.1.2. Interfacial Damping and Strength Indicator

The interfacial damping (tan δ_i_) parameter measures the adhesion between fiber and matrix in terms of matrix volume fraction and ratio of elastic modulus of the matrix and composite (H), which can be written as [58]:

(20) $$\tan\delta_{i}=(\alpha -V_{m}\;H)\;{\left(\tan\delta_{m}\right)}_{\max}$$

where $\alpha =\frac{{\left(\tan\delta_{c}\right)}_{\max}}{\;{\left(\tan\delta_{m}\right)}_{\max}}\;$; ${\left(\tan\delta_{c}\right)}_{\max}$ and $\;{\left(\tan\delta_{m}\right)}_{\max}$ are the maximum loss tangent values of composite and matrix, respectively; $V_{m}$ is the matrix volume fraction = 1 − ${\;V}_{f}$; $V_{f}$ is the fiber volume fraction; $H=\frac{S_{m}}{S_{c}}$; S_m_ and S_c_ are the matrix and composite Young’s moduli, respectively, obtained from the uniaxial tensile test. Higher the value of α, better would be the interfacial damping. The interfacial strength indicator $\sigma_{i}$ can be written in terms of the matrix volume fraction, and the maximum loss tangent values of composite and matrix [47], which is given by:

(21) $$\sigma_{i}=\frac{1-\;\alpha }{\left(1-V_{m}\right)}$$

## 4. Results and Discussions

### 4.1. Mechanical Properties

**Figure 4:**
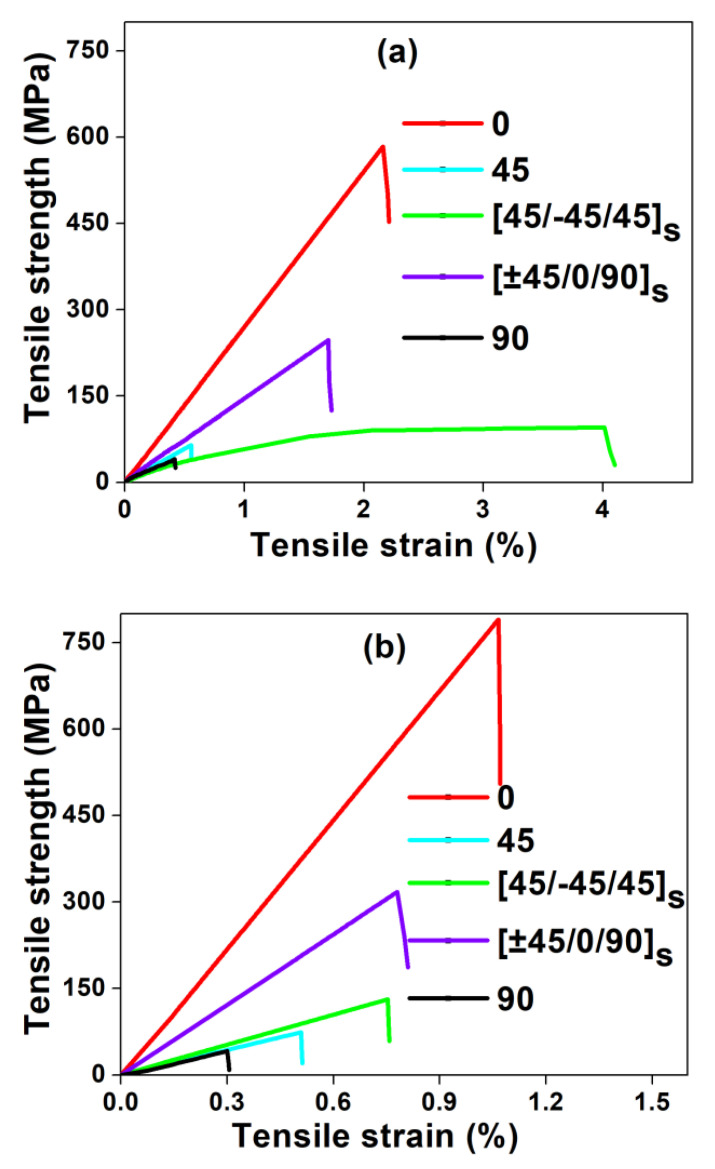
Tensile stress-strain curves of composites: (**a**) GFRP and (**b**) CFRP.

**Figure 5:**
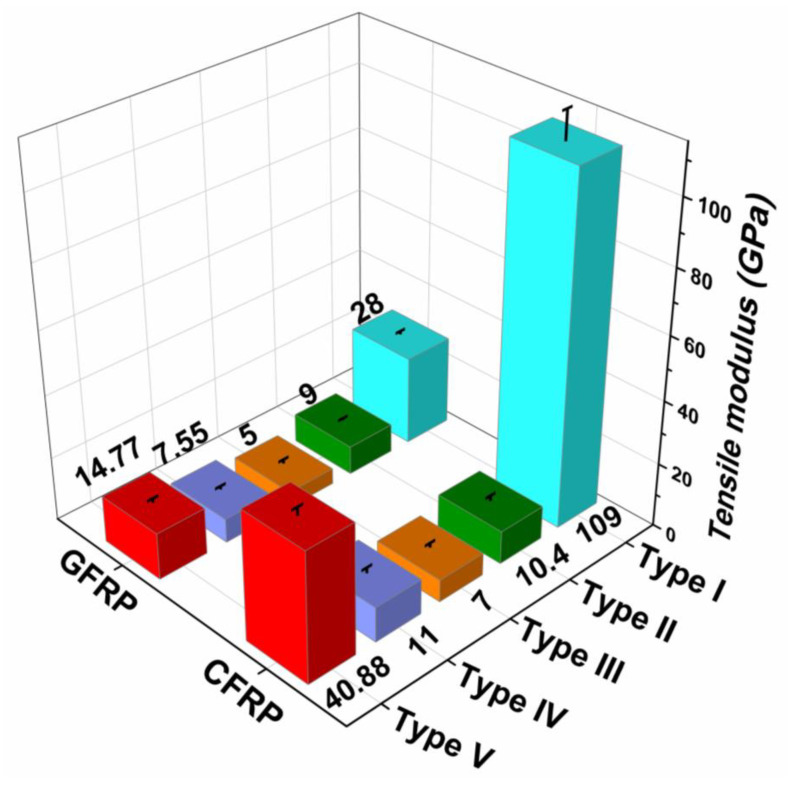
Average tensile modulus for different fiber orientations of GFRP and CFRP composites.

**Figure 6:**
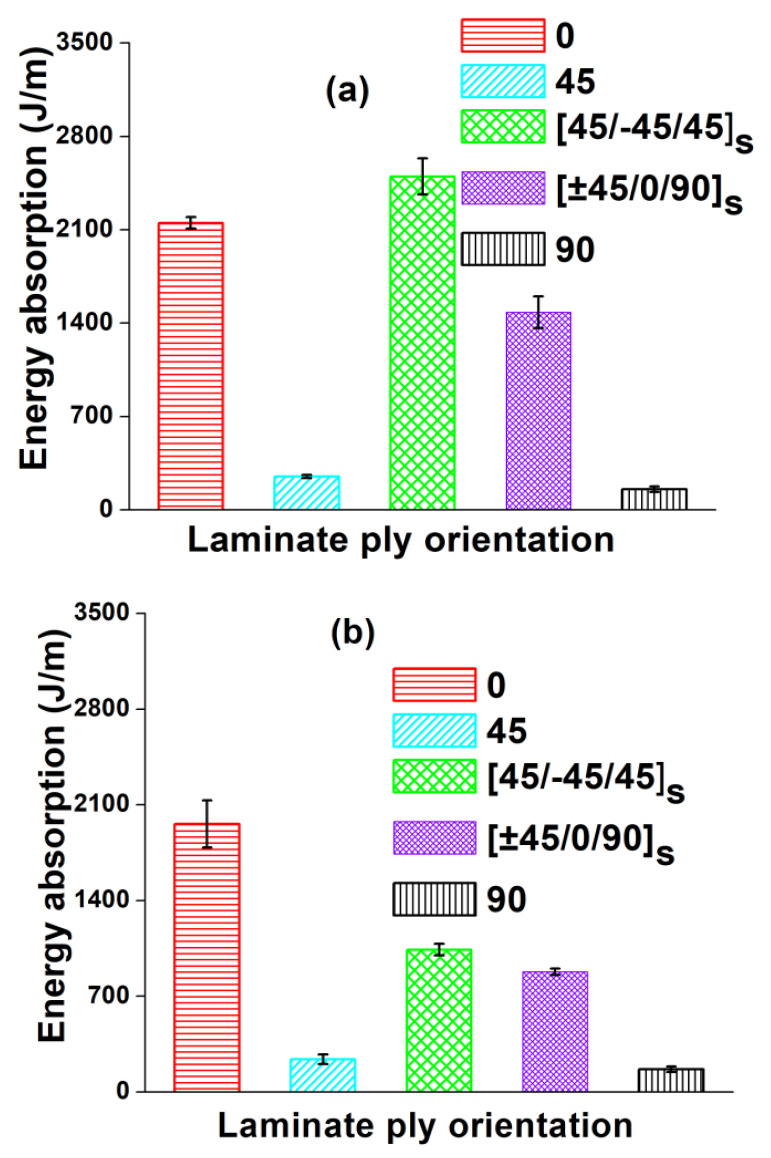
Energy absorption per unit thickness for different fiber orientations of composites: (**a**) GFRP and (**b**) CFRP.

The comparison between flexural, tensile and impact properties of different fiber orientations of glass/epoxy and carbon/epoxy composites are shown in Figure 3, Figure 4, Figure 5 and Figure 6, respectively. In Figure 3, the flexural stress-strain traces indicate the combination of brittle and ductile modes of failure. In contrast, from Figure 4, a sudden drop in stress is observed in the tensile stress-strain curves after reaching the maximum stress, which indicates the brittle failure. The magnitude of flexural strength and flexural strain (%) values are higher in all fiber oriented glass/epoxy and carbon/epoxy composite specimens compared to their tensile strength and tensile strain (%) values. However, it is observed from Figure 5 and Table 1 and Table 2 that the average tensile modulus values are higher in all fiber oriented glass/epoxy and carbon/epoxy composite specimens compared to their average flexural modulus values. This could be due to the difference in loading direction in both tests. 

In contrast to single failure mode (brittle failure) in tensile loading, a combination of compression and tension modes of failure occurs in composites during flexural loading. Three variations are observed in the flexural stress-strain curves: (1) a linear variation in the stress and strain (%) until the maximum stress is reached; (2) a sudden drop in the stress due to initiation of the failure which can be fiber-matrix interface cracking. Besides, slight reductions in the stress with the further increase in the strain (%), due to progressive failure of composite layers; (3) plateau region.

It is worth observing from Figure 3a,b that the degree of non-linearity in the flexural stress-strain curves after reaching the maximum stress is lower for GFRP composites compared to CFRP composites. This indicates that the failure in all laminate configurations of CFRP composites is more brittle compared to that in GFRP composites. As a result, the strain (%) is higher in all laminate configurations of GFRP composites compared to that in CFRP composites whereas strength and modulus are found to be higher in CFRP composites compared to that in GFRP composites. 

It is observed that the (45°/−45°/45°)_s_ (Type IV) glass/epoxy laminate has the highest %strain in both flexural and tensile test cases amongst the other combinations. As a consequence, (45°/−45°/45°)_s_ glass/epoxy laminate possesses the highest energy absorption [Figure 6a]. Hence, the newly proposed laminate can be recommended for crashworthiness applications. 

The tensile strength, strain and modulus are higher in the case of (0°) (Type I) carbon/epoxy laminate than in the case of (45°/−45°/45°)_s_ (Type IV) carbon/epoxy laminate. Therefore, this leads to slightly lower energy absorption characteristics in (45°/−45°/45°)_s_ laminate compared to that in (0°) laminate. [Figure 6b]. However, both laminate configurations possess excellent mechanical properties, followed by QI laminate. The QI (Type V) laminate possesses combined properties, such as taking higher %elongation from (±45°) plies and higher strength and stiffness from 0° plies. On the contrary, (90°) (Type III) laminate possesses the least properties compared to other laminates, due to the matrix dominated failure.

### 4.2. Dynamic Mechanical Analysis

**Figure 7:**
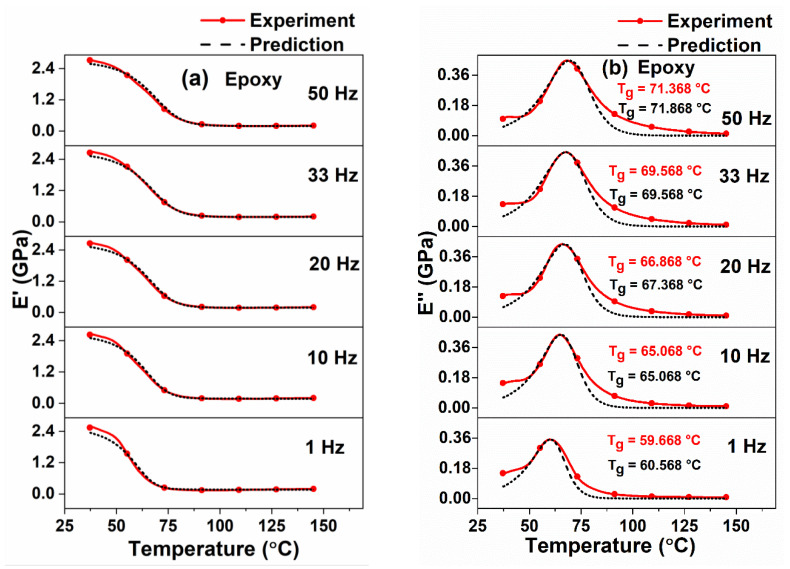
Comparison between predicted and experimental results for different frequencies of neat epoxy resin: (**a**) storage modulus and (**b**) loss modulus.

**Figure 8:**
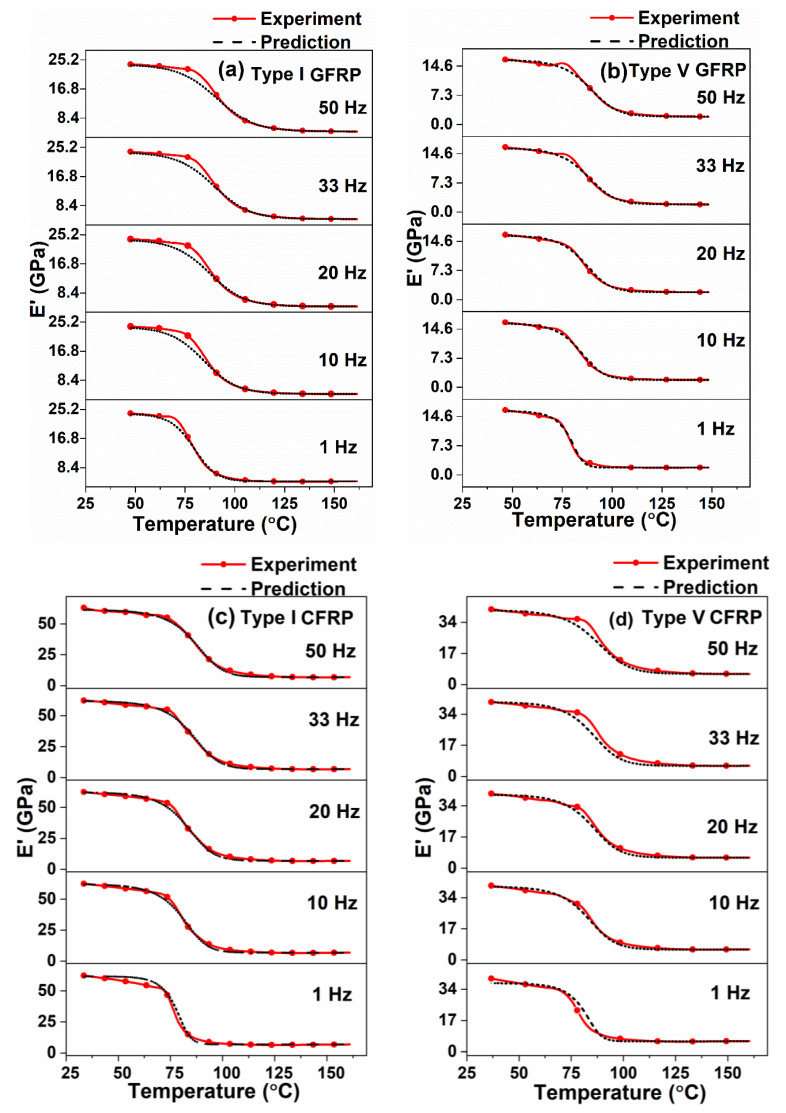
Comparison of predicted storage modulus values with the experimental values for different frequencies: (**a**) Type I GFRP, (**b**) Type V GFRP, (**c**) Type I CFRP and (**d**) Type V CFRP.

**Figure 9:**
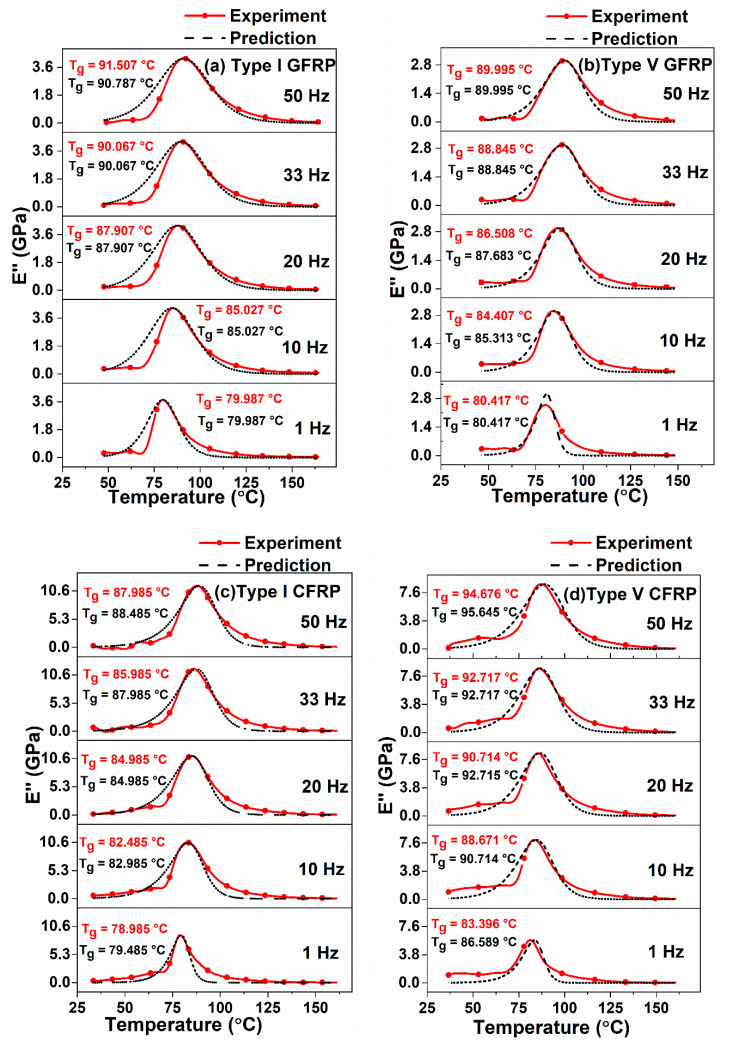
Comparison of predicted loss modulus values with the experimental values for different frequencies: (**a**) Type I GFRP, (**b**) Type V GFRP, (**c**) Type I CFRP and (**d**) Type V CFRP.

In this section, the viscoelastic properties of epoxy and different types of unidirectional GFRP and CFRP composites subjected to different frequencies ranging from 1 Hz to 50 Hz over the temperature range from room temperature to 160 °C are presented. Predicted storage modulus and loss modulus values for the corresponding temperature and frequency ranges were also determined using Equations (16) and (17), respectively. The predicted loss tangent values were determined from the theoretical storage and loss moduli values. The viscoelastic parameters L(f) and S(f) were obtained by curve fitting $\alpha_{T_{g}}$ vs. T [Equation (14)], using MATLAB. The decrease in L(f) and S(f) values with the increase in frequency was observed and values given in Table 1 and Table 2. A similar kind of trend was observed elsewhere [35]. The predicted T_g_ values were obtained from modeling curves of $E_{i}^{\prime \prime }$ and tanδ. An excellent correlation was obtained between the experimental and predicted results, which are shown in Figure 7, Figure 8 and Figure 9. 

It was observed from Figure 7a,b and Table 1, that the difference between the experimental and predicted storage and loss moduli and T_g_ values of neat epoxy samples were less than 10%, 5% and 1.5%, respectively. It was also observed from Figure 7a and Table 1, that the experimental (E’)_max_ increases from 2.572 GPa at 1 Hz to 2.715 GPa at 50 Hz (5.56% increase) whereas, the predicted (E’)_max_ increases from 2.350 GPa at 1 Hz to 2.580 GPa at 50 Hz (9.79%). A reason for this increment in the storage modulus in higher frequencies is due to less availability of time for rearrangement of molecules in the resin than in the lower frequencies [13,59]. Similarly, for the same frequency range of 1 HZ and 50 Hz, the increase in experimental and predicted T_g_ values were observed from Figure 7b that the difference being 19.61% and 18.66%, respectively. This shows excellent agreement between the experimental and predicted results. A similar kind of trend (increase in storage modulus and T_g_ values with the increase in frequency) was observed for different fiber orientations of GFRP and CFRP composites from Figure 8 and Figure 9 and Table 1 and Table 2. 

It can be observed from Figure 7a and Figure 8a–d that the storage modulus decreases dramatically, when the temperature exceeds the T_g_, mainly owing to material softening. The temperature at which the storage modulus starts to decrease is low for epoxy samples compared to FRPCs. This can be attributed to an increase in rigidity of the structure when fibers are added in the epoxy resin. 

Further observations can be made from Figure 7b and Figure 9 that initially the loss modulus curve is slightly horizontal, which is mainly due to immobility of molecules in the polymer matrix. For these low temperatures, there will not be any translational and rotational movements occurring in the molecular chains. As the temperature increases, the molecules start moving in the resin, which leads to a change in the shape of the curve. In contrast to storage modulus, the loss modulus curve increases up to T_g_. After reaching the T_g_, the resistance to molecular motion decreases, due to a combination of translational and rotational motion of molecules in the resin.

Adverse effects occur at elevated temperatures which are due to softening of the resin, as there is almost no control over molecular transport phenomena in the matrix. Consequently, the curve starts to decrease up to the rubbery region, where a plateau-shaped distribution is seen. This drop-in loss modulus can be minimized by stiffening the structure through the incorporation of fibers. Figure 9 indicates the higher loss modulus values of FRPCs compared to the loss modulus values of the neat epoxy samples seen in Figure 7b.

#### 4.2.1. The Effect of Frequency on Storage Modulus (E’)

**Figure 10:**
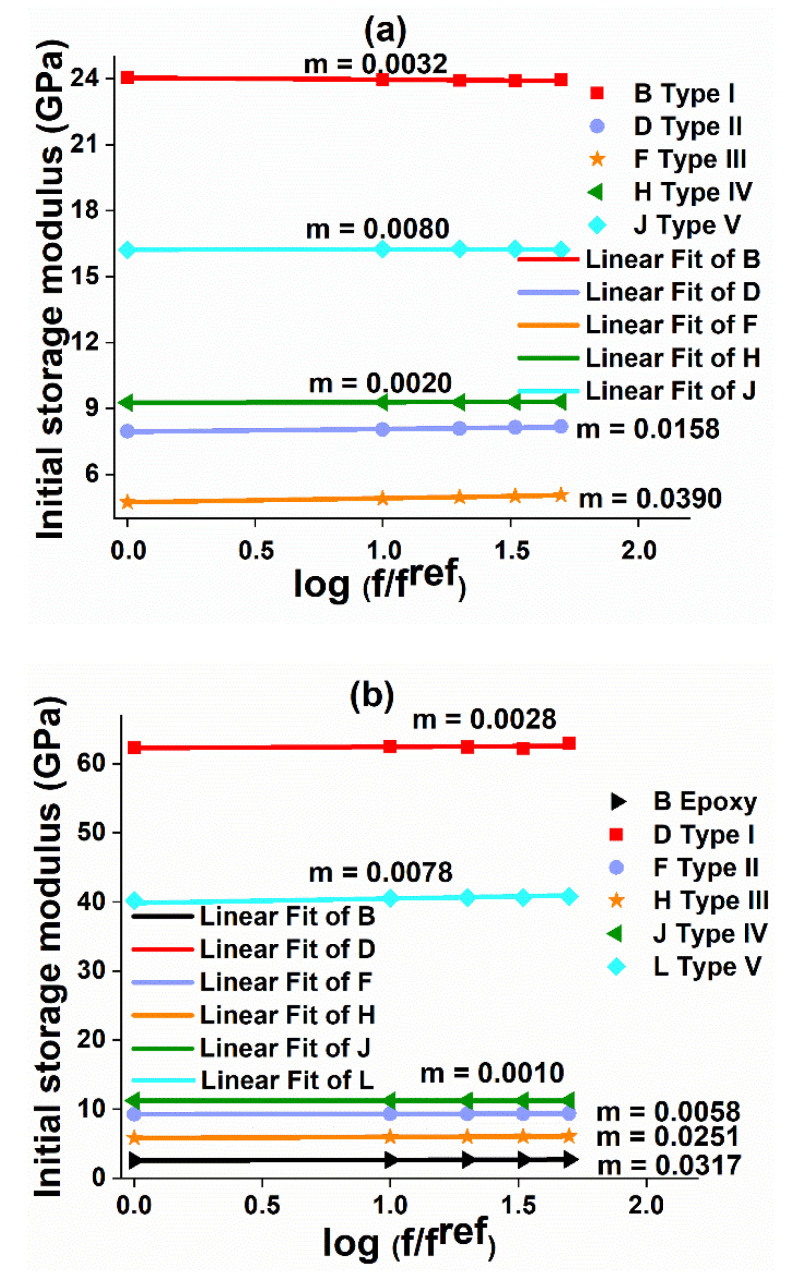
Frequency sensitivity coefficient: (**a**) GFRP and (**b**) epoxy and CFRP composite.

Investigating the frequency effects on storage modulus is essential, which provides the vibrational response of the structure during dynamic loading conditions. The frequency sensitivity coefficient (m) is determined by a linear curve fitting procedure using Equation (7), to determine the frequency effects on the initial storage modulus of neat epoxy and different fiber orientations of CFRP and GFRP samples, which are shown in Figure 10a,b, respectively. The higher the sensitivity coefficient, the lower the resistance to cyclic loading of the structure during service life.

**Figure 11:**
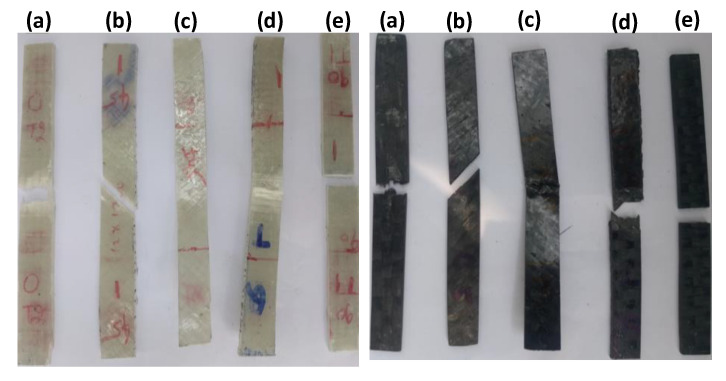
Fractured surfaces after flexural testing of glass/epoxy and carbon/epoxy composite specimens for different fiber orientations: (**a**) 0, (**b**) 45, (**c**) (45/−45/45)s, (**d**) (±45/0/90)s and (**e**) 90.

**Figure 12:**
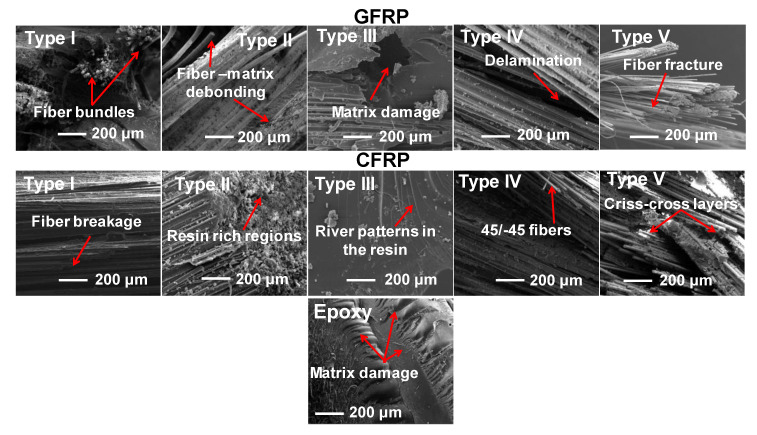
SEM micrographs of the fractured surface of composites and epoxy subjected to three-point bending.

Figure 10 confirms that the sensitivity coefficient is higher for epoxy samples followed by Type III laminates in both CFRP and GFRP samples. The neat epoxy and Type III laminates fail rapidly due to their weaker structure indicated by higher m values. The fewer amount of fibers underneath the three-point bending fixture in Type III laminate (Figure 11e and Figure 12) lead to higher m values compared to the composites of different fiber orientations. Therefore, these structures are not recommended for applications where dynamic loading (e.g., gust wind or turbulence) situations predominantly occur. The stiffer CFRP composites have lower m values compared to GFRP composites. In both GFRP and CFRP composites, Type IV laminates exhibit lower m values. This emphasizes that Type IV laminates possess more stable laminate configuration, which can bear cyclic loads effectively to a larger extent, and can perform better in vibration isolation structure for any drive-shaft, due to higher flexural strains offered by ±45° plies. 

Figure 11 (fractured samples) and Figure 12 (SEM micrographs) show that laminates having layers of different ply angles demonstrate a strong interlocking effect compared to laminates with layers of identical ply angles. In particular, 45° (Type II) and 90° (Type III) laminates show weaker networks due to matrix dominated failures. Higher bending strain could be possible in laminates of different plies (Type IV and Type V) compared to laminates with identical plies (Figure 3a,b). As a result, the crack propagation can be delayed in these composites and therefore, catastrophic failure is not occurred [40]. This is due to the criss-cross fiber patterns presented in Type IV and Type V laminates observed from the fractured surfaces of glass/epoxy and carbon/epoxy composites in Figure 11c,d and can also be seen from SEM micrographs (Figure 12).

Table 1 and Table 2 show that average flexural modulus values correlate very well with the experimental and predicted storage moduli values of neat epoxy and different laminate types of GFRP and CFRP samples. Amongst all types, Type I laminates exhibited higher flexural modulus and (E’)_max_ values in both GFRP and CFRP composites, while neat epoxy resin samples exhibited lower flexural and storage moduli values. This behavior is attributed to long and aligned fibers offering more resistance to deformation, which can be confirmed from fractured specimens and SEM micrographs illustrated in Figure 11 and Figure 12, respectively.

#### 4.2.2. Experimental and Predicted Loss Tangent and Corresponding T_g_

**Figure 13:**
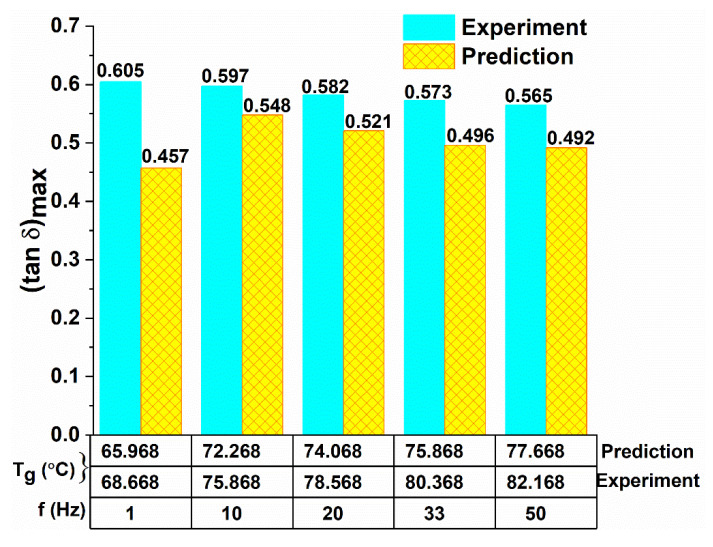
Comparison of predicted loss tangent and its T_g_ values with experimental results for different frequencies of epoxy.

**Figure 14:**
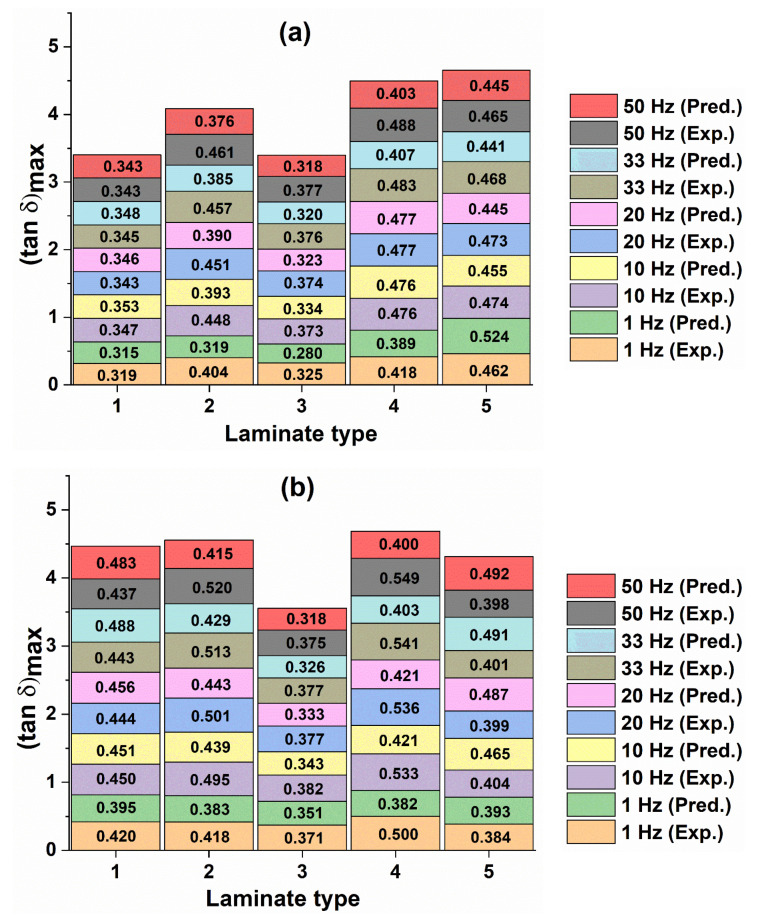
Comparison of predicted loss tangent values with experimental results for different types and frequencies of GFRP (**a**) and CFRP (**b**) composites.

**Figure 15:**
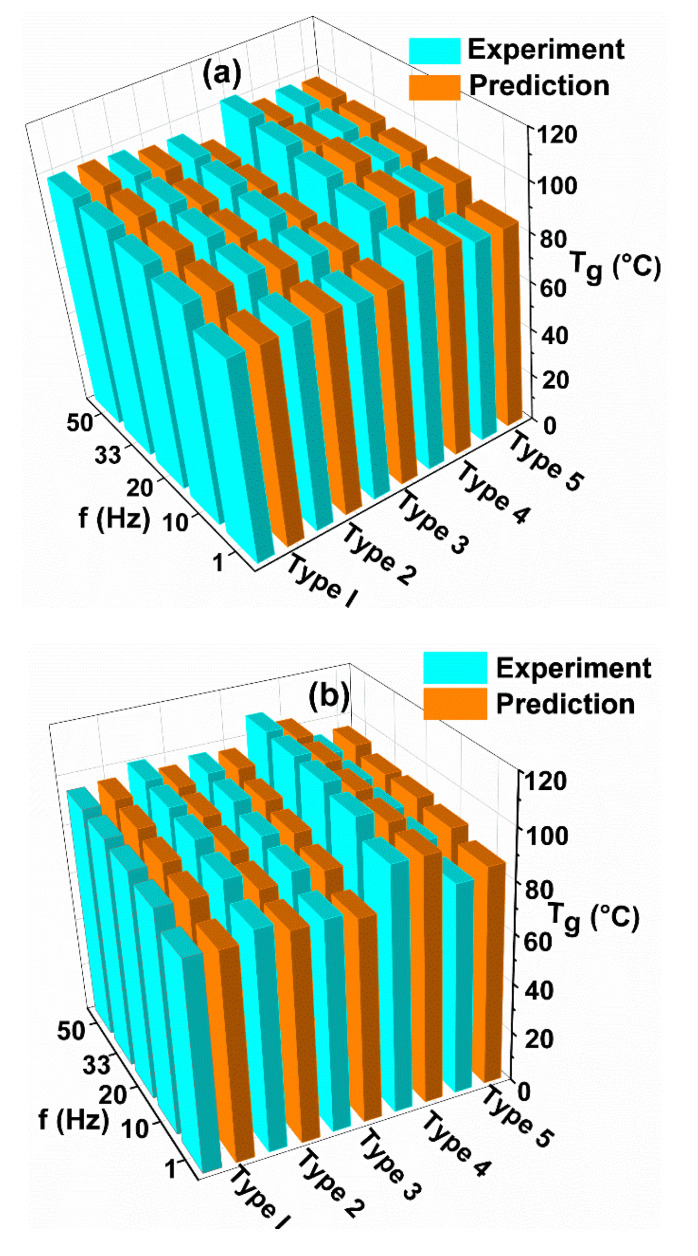
Comparisons of predicted T_g_ [(tan δ)_max_] values with experimental results for different types and frequencies of GFRP (**a**) and CFRP (**b**) composites.

Figure 13, Figure 14 and Figure 15 show the experimental and predicted (tan δ)_max_ values and their corresponding peak temperature values, taken as T_g_ values for neat epoxy and different types of GFRP and CFRP composites, respectively. These figures indicate a reasonably good agreement between the experimental and predicted values. It is observed from Figure 14 that the (tan δ)_max_ values are lower for composites compared to epoxy (Figure 13), in all frequencies. The decrease in (tan δ)_max_ values is due to the incorporation of fibers in the matrix as the storage modulus of the structure increases by the addition of fibers. The loss tangent is inversely proportional to the storage modulus from Equation (2). Similar observations were made in [30,47]. 

However, the T_g_ values increase significantly when the fibers are introduced in epoxy resin. In particular, Type IV laminates in both GFRP and CFRP samples showed higher T_g_ values compared to other laminate configurations as shown in Figure 15a,b. A similar trend was observed in both experimental and predicted values. The substantial increase in T_g_ due to the incorporation of fibers in the epoxy matrix was also observed elsewhere [12,13].

Figure 13 shows the T_g_ ((tan δ)_max_) increases from 68.668°C at 1 Hz to 82.168 °C at 50 Hz for neat epoxy samples (19.66% increase). Similarly, the predicted T_g_ ((tan δ)_max_) value increases from 65.968 °C at 1 Hz to 77.668 °C at 50 Hz for neat epoxy samples (17.74% increase). 

In general, the T_g_ value cannot be fixed for FRPCs, there should be some range given. The fiber orientation effect can play a significant role to attain maximum T_g_ value and a range. There is an approximately 10 °C difference obtained from different fiber oriented samples by Coban et al. [60]. Similarly, in the present work, the deviation in T_g_ values between different fiber orientated samples of GFRP and CFRP composites of frequency range 1 Hz and 50 Hz obtained was less than or equal to 10 °C (Figure 13). Since all samples were fabricated using the same epoxy resin. 

In Figure 15, the increase in T_g_ value with the increase in frequency from 1 Hz to 50 Hz was also observed in all samples, as similar to the trend seen in the epoxy matrix in Figure 13. This is due to an increase in the speed of the test as the frequency is directly proportional to the test speed and inversely proportional to the molecular relaxation time. The deviation between the minimum T_g_ value at the frequency of 1 Hz and the maximum T_g_ value at the frequency of 50 Hz, obtained was less than or equal to 15 °C (Figure 15) in GFRP and CFRP composites. Similar results were found by Bergent et al. [37] for different fiber orientations of woven CFRP composites. The experimental and predicted T_g_ values are in good agreement.

#### 4.2.3. The Effect of Frequency on T_g_

**Figure 16:**
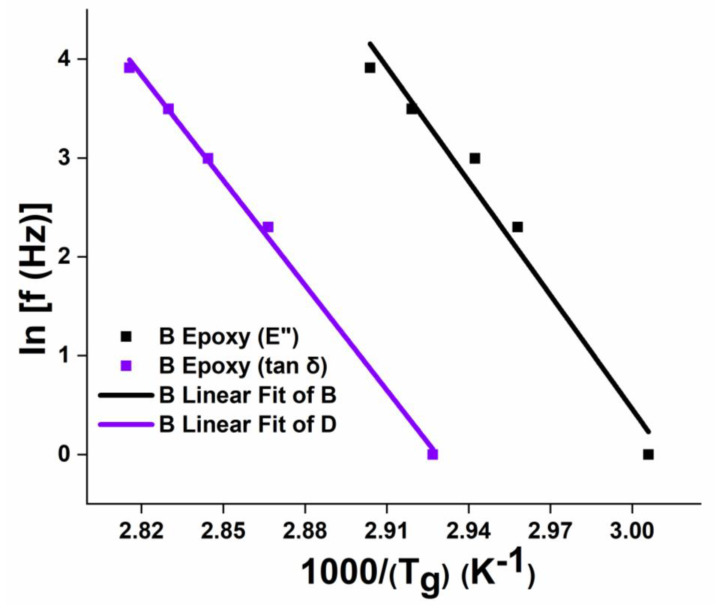
Effect of frequency on T_g_ ((E”)_max_) and T_g_ ((tan δ)_max_) of epoxy.

**Figure 17:**
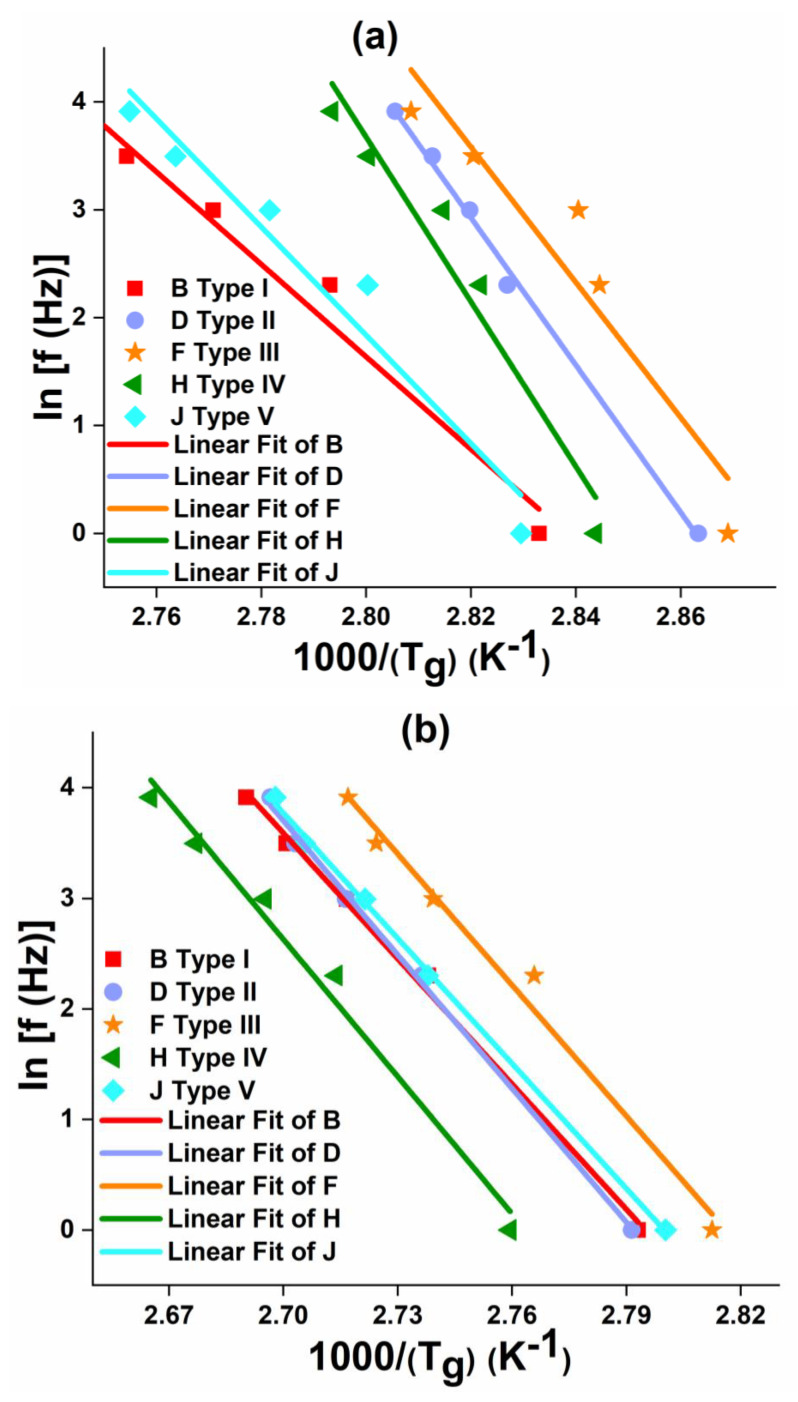
Effect of frequency on T_g_ of GFRP composites: (**a**) loss modulus and (**b**) Loss tangent.

**Figure 18:**
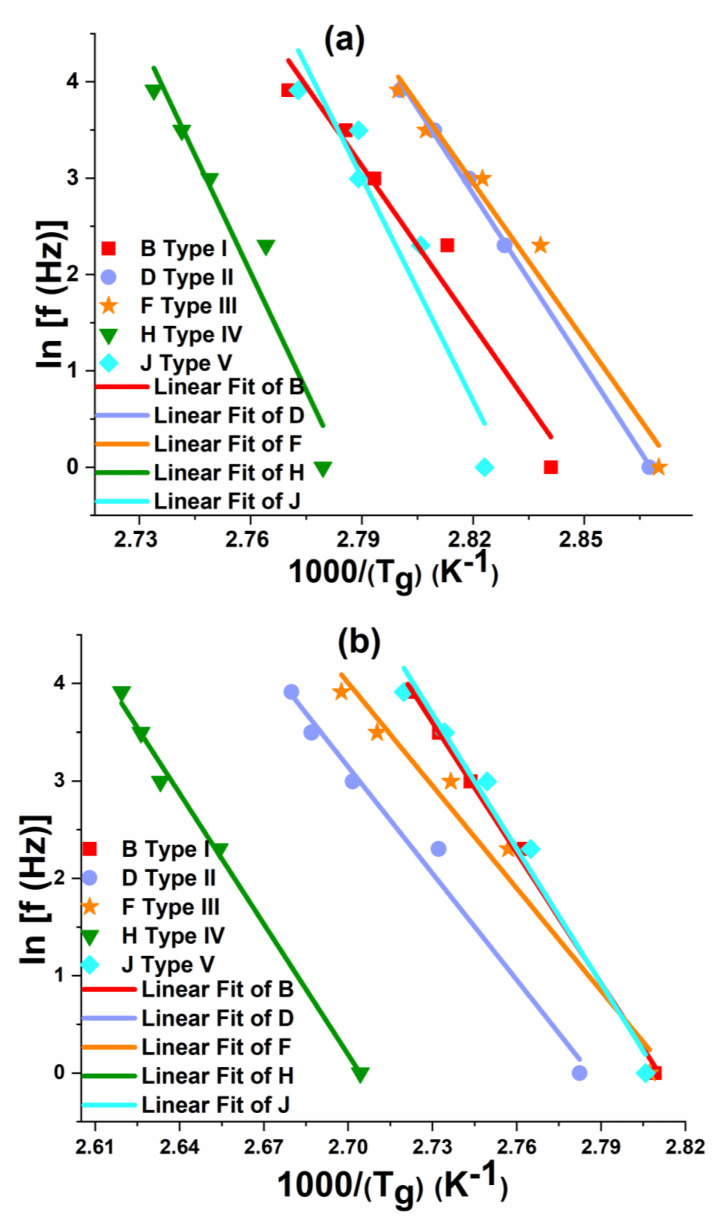
Effect of frequency on T_g_ of CFRP composites: (**a**) loss modulus and (**b**) Loss tangent.

Figure 16, Figure 17 and Figure 18 show the variation in T_g_ values corresponding to (E’’)_max_ and (tan δ)_max_ with the increase in frequency (f) from 1 Hz to 50 Hz for neat epoxy resin, GFRP and CFRP composites, respectively. From these figures, it is worth noting that T_g_ values increased with increasing frequency. These curves show a slope: $\ln\;\left(f\right)\;\mathrm{vs}.\;1/\left(T_{g}\right)$ for estimating the activation energy using Equation (4) through the Arrhenius model. Higher values (~>0.9) of the coefficient of determination (R^2^) are obtained, which confirm an excellent correlation between the experimental values and the curve fit.

It was observed from Figure 16, Figure 17 and Figure 18 that the FRPCs have larger slopes compared to neat epoxy resin samples, as a result, higher activation energy values were obtained, which are shown in Table 3 and Table 4. In particular, Type IV laminates have higher activation energy values compared to other laminates. The activation energy corresponding to (E”)_max_ and (tan δ)_max_ of Type IV CFRP laminates were 6.6% and 8% higher respectively, as compared to corresponding activation energy values of Type IV GFRP laminates. These results emphasize that the higher thermal stability of Type IV laminates can be a better choice for future aerospace and other high-temperature applications. It was also observed from Table 3 and Table 4 that (E_a_)_(E”)max_ values are higher than (E_a_)_(tan δ)max_ values in neat epoxy and FRPCs. Similar kinds of observations were found elsewhere for different fiber orientations of woven roving composites [13].

#### 4.2.4. The Effect of Frequency on Strength Indicator and Interfacial Damping 

The strength indicator $\left(\sigma_{i}\right)$ and interfacial damping ($\tan\delta_{i})$ are essential parameters which provide the information related to interfacial strength and bonding between fibers and matrix. These parameters rely mostly on matrix properties. $\sigma_{i}$ and $\tan\delta_{i}$ are determined using Equations (20) and (21), respectively, and are given in Table 5. The decrease in $\sigma_{i}$ and an increase in $\tan\delta_{i}$, with the increasing frequency are observed from the table. However, these values depend on fiber and matrix volume fractions. The decrease in $\sigma_{i}$ with the increase in frequency, is less for Type IV CFRP, followed by Type I GFRP composites compared to other laminate configurations. 

## 5. Conclusions

In this study, static and dynamic mechanical properties of neat epoxy and different laminate configurations of GFRP and CFRP composites were investigated and compared. The novel approach is made for predicting the temperature-frequency dependent viscoelastic properties of neat epoxy and composite panels. The obtained results show a good agreement between the predicted and experimental values. The T_g_ values obtained from the experimental results and modeling curves, agree reasonably well. An excellent correlation between static flexural modulus and frequency-dependent initial storage modulus values was obtained. Further, the strength indicator and interfacial damping parameters were determined using both static and dynamic mechanical properties.

The neat epoxy samples exhibit high loss tangent and low T_g_ and activation energy values compared to the composite laminates. Type I CFRP laminates exhibit high storage modulus values, whereas Type IV CFRP laminates exhibit high T_g_ and activation energy values compared to other laminate configurations. Storage modulus values of Type IV laminates were less sensitive to frequency compared to neat epoxy and other laminate configurations. These important findings are very useful for dynamic properties of composite structures such as, GVT and the designing and manufacturing of automobile drive shafts. In particular, Type IV laminates are recommended for vibration isolation applications, owing to their better impact energy absorption capabilities, higher activation energy and T_g_ values, and lower frequency sensitivity coefficient. It can also be a better material for composite structures, which are exposed to harsh loading conditions. Also, the experimental data presented in this study will be useful to develop artificial neural network-based viscoelastic models.

## Figures and Tables

**Table 1:** The effect of frequency on initial storage modulus of epoxy and glass/epoxy composites.

Material	Frequency (Hz)	Parameters		Initial Storage Modulus (GPa) (Experiment)	Initial Storage Modulus (GPa) (Prediction)	%error	Flexural Modulus (GPa)
		L(f)	S(f)				
**Epoxy**	1	0.0875	2.941	2.572	2.350	9.447	
	10	0.087	2.530	2.652	2.503	5.953	
	20	0.085	2.278	2.673	2.518	6.156	2.85 ± 0.07
	33	0.0843	2.124	2.692	2.527	6.529	
	50	0.0839	2.118	2.715	2.580	5.233	
	**Avg**			**2.660 ± 0.054**	**2.4956 ± 0.086**		
**TYPE I**	1	0.150	1.250	24.052	23.797	1.072	26.25 ± 0.6
	10	0.109	1.119	23.935	23.535	1.699	
	20	0.105	1.110	23.917	23.543	1.589	
	33	0.102	1.109	23.915	23.540	1.593	
	50	0.099	1.090	23.934	23.638	1.252	
	**Avg**			**23.950 ± 0.057**	**23.610 ± 0.112**		
**TYPE II**	1	0.172	1.560	7.950	7.907	0.543	8.11 ± 0.57
	10	0.134	1.525	8.043	7.349	9.443	
	20	0.129	1.514	8.082	7.379	9.527	
	33	0.115	1.510	8.132	7.413	9.700	
	50	0.095	1.50	8.177	7.429	10.00	
	**Avg**			**8.076 ± 0.087**	**7.495 ± 0.232**		
TYPE III	1	0.110	2.490	4.737	4.256	11.301	4.45 ± 0.63
	10	0.096	2.440	4.904	4.367	12.297	
	20	0.0955	2.438	4.964	4.413	12.486	
	33	0.095	2.437	5.012	4.453	12.553	
	50	0.094	2.436	5.058	4.488	12.700	
	**Avg**			**4.935 ± 0.124**	**4.395 ± 0.1**		
**TYPE IV**	1	0.094	2.497	9.269	8.868	4.522	7.67 ± 0.29
	10	0.093	2.482	9.283	8.894	4.374	
	20	0.0929	2.441	9.284	8.915	4.139	
	33	0.0927	2.432	9.296	8.931	4.087	
	50	0.0925	2.430	9.305	8.956	3.897	
	**Avg**			**9.287 ± 0.013**	**8.913 ± 0.033**		
**TYPE V**	1	0.159	2.75	16.117	15.937	1.129	14.31 ± 1.84
	10	0.125	1.579	16.258	15.942	1.982	
	20	0.120	1.550	16.348	15.984	2.278	
	33	0.110	1.499	16.458	15.922	3.366	
	50	0.109	1.497	16.223	16.036	1.166	
	**Avg**			**16.281 ± 0.129**	**15.964 ± 0.046**		

**Table 2:** The effect of frequency on initial storage modulus of carbon/epoxy composites.

Material	Frequency (Hz)	Parameters		Initial Storage Modulus (GPa) (Experiment)	Initial Storage Modulus (GPa) (Prediction)	%error	Flexural Modulus (GPa)
		L(f)	S(f)				
**TYPE I**	1	0.165	2.461	62.332	61.801	0.859	73.75 ± 1.9
	10	0.101	2.214	62.500	62.001	0.805	
	20	0.099	1.975	62.413	62.097	0.509	
	33	0.098	1.953	62.232	61.741	0.795	
	50	0.097	1.939	62.948	61.187	2.878	
	**Avg**			**62.485 ± 0.277**	**61.765 ± 0.354**		
**TYPE II**	1	0.099	2.65	9.277	8.671	6.989	10.5 ± 0.7
	10	0.092	2.567	9.314	8.748	6.470	
	20	0.087	2.548	9.331	8.830	5.674	
	33	0.086	2.298	9.346	8.925	4.717	
	50	0.085	2.15	9.380	8.979	4.466	
	**Avg**			**9.330 ± 0.038**	**8.831 ± 0.126**		
**TYPE III**	1	0.105	3.557	5.837	5.377	8.555	6 ± 0.77
	10	0.073	3.154	5.972	5.784	3.250	
	20	0.059	3.125	6.006	5.722	4.963	
	33	0.055	2.990	6.059	5.760	5.191	
	50	0.050	2.550	6.095	5.411	12.641	
	**Avg**			**5.994 ± 0.099**	**5.611 ± 0.199**		
**TYPE IV**	1	0.075	2.870	11.234	10.396	8.061	9.5 ± 0.86
	10	0.074	2.849	11.233	10.441	7.5851	
	20	0.072	2.829	11.237	10.447	7.562	
	33	0.0719	2.480	11.230	10.457	7.392	
	50	0.071	2.469	11.272	10.501	7.342	
	**Avg**			**11.241 ± 0.017**	**10.448 ± 0.037**		
**TYPE V**	1	0.130	2.799	40.233	37.324	7.794	38.53 ± 1.17
	10	0.106	1.679	40.533	39.902	1.581	
	20	0.105	1.667	40.658	39.958	1.752	
	33	0.104	1.658	40.627	40.434	0.477	
	50	0.094	1.598	40.827	40.401	1.054	
	**Avg**			**40.576 ± 0.219**	**39.604 ± 1.298**		

**Table 3:** Activation energy of epoxy and glass/epoxy composites.

Material	Frequency (Hz)	T_g_ ((E^”^)_max_)(°C) (Experiment)	(E_a_)_(E”)max_ (kJ/mol)	T_g_ ((tan δ)_max_)(°C) (Experiment)	(E_a_)_(tan δ)max_ (kJ/mol)
Epoxy	1	59.668	319.598	68.668	294.332
	10	65.068		75.868	
	20	66.868		78.568	
	33	69.568		80.368	
	50	71.368		82.168	
TYPE I	1	79.987	356.346	85.027	314.310
	10	85.027		92.227	
	20	87.907		95.107	
	33	90.067		97.267	
	50	91.507		98.707	
TYPE II	1	76.241	567.713	85.241	
	10	80.741		92.441	
	20	81.641		95.141	335.445
	33	82.541		96.941	
	50	83.441		97.841	
TYPE III	1	75.555	521.271	82.555	
	10	78.555		88.555	
	20	79.055		92.055	328.577
	33	81.555		94.055	
	50	83.055		95.055	
TYPE IV	1	78.646		89.386	
	10	81.380		95.484	
	20	82.285	634.865	98.025	344.207
	33	84.085		100.541	
	50	84.977		102.202	
TYPE V	1	80.417	417.196	84.107	
	10	84.107		92.245	
	20	86.508		94.453	313.861
	33	88.845		96.601	
	50	89.995		97.659	

**Table 4:** Activation energy of carbon/epoxy composites.

Material	Frequency (Hz)	T_g_ ((E^”^)_max_) (°C) (Experiment)	(E_a_)_(E”)max_ (kJ/mol)	T_g_ ((tan δ)_max_) (°C) (Experiment)	(E_a_)_(tan δ)max_ (kJ/mol)
**TYPE I**	1	78.985	458.691	82.985	370.870
	10	82.485		88.985	
	20	84.985		91.485	
	33	85.985		92.985	
	50	87.985		94.485	
**TYPE II**	1	75.724	492.380	86.403	303.369
	10	80.543		93.003	
	20	81.737		97.148	
	33	82.920		99.166	
	50	84.093		100.160	
**TYPE III**	1	75.421		83.215	291.937
	10	79.337		89.722	
	20	81.290	452.713	92.426	
	33	83.216		95.963	
	50	84.165		97.702	
**TYPE IV**	1	86.774	676.784	96.774	371.935
	10	88.774		103.774	
	20	90.774		106.774	
	33	91.774		107.774	
	50	92.774		108.774	
**TYPE V**	1	81.221		83.396	312.706
	10	83.396		88.671	
	20	85.533	641.134	90.714	
	33	85.535		92.717	
	50	87.634		94.676	

**Table 5:** Strength indicator and interfacial damping parameters of GFRP and CFRP composites.

Frequency (Hz)	GFRP Laminate Type	Fiber Volume Fraction	$\sigma_{i}$	$\tan\delta_{i}$	CFRP Laminate Type	Fiber Volume Fraction		
1 10 20 33 50	Type I	0.487	0.971	0.286	Type I	0.449	0.681	0.411
			0.859	0.314			0.548	0.441
			0.843	0.311			0.528	0.435
			0.817	0.314			0.505	0.434
			0.807	0.312			0.504	0.428
1 10 20 33 50	TYPE II	0.511	0.650	0.305	TYPE II	0.527	0.586	0.335
			0.488	0.351			0.324	0.414
			0.440	0.356			0.264	0.422
			0.396	0.364			0.199	0.435
			0.360	0.369			0.151	0.443
1 10 20 33 50	TYPE III	0.529	0.875	0.154	TYPE III	0.540	0.716	0.252
			0.709	0.204			0.667	0.264
			0.676	0.209			0.652	0.262
			0.650	0.214			0.633	0.264
			0.629	0.217			0.623	0.264
1 10 20 33 50	TYPE IV	0.418	0.739	0.278	TYPE IV	0.491	1.00	0.334
			0.485	0.338			0.951	0.393
			0.437	0.342			0.945	0.396
			0.376	0.350			0.935	0.403
			0.326	0.357			0.918	0.410
1 10 20 33 50	TYPE V	0.40	0.589	0.388	TYPE V	0.415	0.880	0.358
			0.518	0.401			0.779	0.378
			0.467	0.402			0.758	0.374
			0.457	0.398			0.723	0.376
			0.441	0.396			0.712	0.374

## Nomenclature

E’	Storage modulus
$E_{G}^{\prime }$	Glassy storage modulus
$E_{R}^{\prime }$	Rubbery storage modulus
E”	Loss modulus
tan δ	Loss tangent
tan δ_i_	Interfacial damping
T	Temperature
T_g_	Glass transition temperature
$\alpha_{T_{g}}$	Degree of glass transition
f	Frequency
R	Gas constant
E_a_	Activation energy
k	Boltzmann constant
m	Frequency sensitivity coefficient
σ_i_	Strength Indicator
L(f)	Intrinsic growth rate of the glass transition region
S(f)	Symmetry of the glass transition region
V_f_	Fiber volume fraction
